# Construction of a Nomogram Model for Predicting Peritoneal Dissemination in Gastric Cancer Based on Clinicopathologic Features and Preoperative Serum Tumor Markers

**DOI:** 10.3389/fonc.2022.844786

**Published:** 2022-06-01

**Authors:** Dandan Bao, Zhangwei Yang, Senrui Chen, Keqin Li, Yiren Hu

**Affiliations:** ^1^ Department of General Surgery, The Third Clinical Institute Affiliated to Wenzhou Medical University, The Third Affiliated hospital of Shanghai University, Wenzhou People’s Hospital, Wenzhou, China; ^2^ Department of General Surgery, Medical College of Soochow University, Soochow, China

**Keywords:** gastric cancer, peritoneal dissemination, prediction nomogram, serum tumor markers, risk factors

## Abstract

**Background:**

Peritoneal dissemination (PD) is the most common mode of metastasis for advanced gastric cancer (GC) with poor prognosis. It is of great significance to accurately predict preoperative PD and develop optimal treatment strategies for GC patients. Our study assessed the diagnostic potential of serum tumor markers and clinicopathologic features, to improve the accuracy of predicting the presence of PD in GC patients.

**Methods:**

In our study, 1264 patients with GC at Fudan University Shanghai Cancer Center and Wenzhou people’s hospital from 2018 to 2020 were retrospectively analyzed, including 316 cases of PD and 948 cases without PD. All patients underwent enhanced CT scan or magnetic resonance imaging (MRI) before surgery and treatment. Clinicopathological features, including tumor diameter and tumor stage (depth of tumor invasion, nearby lymph node metastasis and distant metastasis), were obtained by imaging examination. The independent risk factors for PD were screened through univariate and multivariate logistic regression analyses, and the results were expressed with 95% confidence intervals (CIs). A model of PD diagnosis and prediction was established by using Cox proportional hazards regression model of training set. Furthermore, the accuracy of the prediction model was verified by ROC curve and calibration plots.

**Results:**

Univariate analysis showed that PD in GC was significantly related to tumor diameter (odds ratio (OR)=12.06, p<0.0006), depth of invasion (OR=14.55, p<0.0001), lymph node metastases (OR=5.89, p<0.0001), carcinoembryonic antigen (CEA) (OR=2.50, p<0.0001), CA125 (OR=11.46, p<0.0001), CA72-4 (OR=4.09, p<0.0001), CA19-9 (OR=2.74, p<0.0001), CA50 (OR=5.20, p<0.0001) and CA242 (OR=3.83, p<0.0001). Multivariate analysis revealed that clinical invasion depth and serum marker of CA125 and CA72-4 were independent risk factors for PD. The prediction model was established based on the risk factors using the R program. The area under the curve (AUC) of the receiver operating characteristics (ROC) was 0.931 (95% CI: 0.900–0.960), with the accuracy, sensitivity and specificity values of 90.5%, 86.2% and 82.2%, respectively.

**Conclusion:**

The nomogram model constructed using CA125, CA72-4 and depth of invasion increases the accuracy and sensitivity in predicting the incidence of PD in GC patients and can be used as an important tool for preoperative diagnosis.

## Introduction

Gastric cancer (GC) is one of the most important gastrointestinal malignancies, ranking fifth among all cancers in the world and representing the third leading cause of cancer-related deaths (1). PD is the most common and important mode of gastric cancer metastasis, occurs in greater than 55%-60% of patients with metastatic gastric cancer, leading to poor prognosis (2–5). Patients with PD often have complications of malignant ascites, abdominal distension and intestinal obstruction and exhibit poor overall survival (6–8). According to statistics, the median overall survival (OS) of GC patients with PD is 3 to 4 months (9, 10), and the 5-year survival rate is 2% (11). Relevant guidelines clearly specify that GC patients with PD can only receive palliative care or neoadjuvant chemotherapy instead of radical surgery (12, 13). With the development of intraperitoneal chemotherapy and the application of immunotherapy and targeted therapy, the median OS in GC patients with PD is expected to improve (14). Therefore, the accurate prediction of PD is essential in the treatment strategies of GC patients.

In clinical practice, computed tomography (CT) is a fundamental imaging modality for the diagnosis of PD, exhibits high specificity and accuracy, whereas its sensitivity is low (15). Studies are unanimous in the conclusion that higher CT sensitivity is dependent on the size of peritoneal metastatic nodules (the detection rates ranged from 8–67%), whereas metastatic nodules are usually small, and therefore have a high rate of misdiagnosis (16, 17). Magnetic resonance imaging (MRI) is superior to CT in contrast resolution and imaging ability. However, it is susceptible to motion artifacts associated with respiration and intestinal peristalsis and requires a long scan time. In addition, MRI is limited by its low sensitivity to the detection of small nodules (18). In addition, PET is costly, and early lesions may go undetected. Diagnostic laparoscopic exploration provides direct observation of peritoneal metastases with high accuracy but is an unconventional and invasive procedure. Serum tumor markers (AFP, CEA, CA125, CA72–4, CA242, CA19–9and CA50) are commonly used in the clinic for the diagnosis and prognostic monitoring of GC. For example, CA125 has been widely confirmed to have clinical guiding value in PD diagnosis in recent years, but the sensitivity and specificity of individual indicators are low (19, 20). Accurate preoperative prediction of PD not only avoids unnecessary invasive operations but also provides opportunities for early comprehensive treatment, which has important clinical value and application prospects. Therefore, we conducted a retrospective study to explore a highly sensitive and noninvasive predictor.

## Materials and Methods

We collected 1498 patients diagnosed with gastric cancer at Fudan University Shanghai Cancer Center (FUSCC) and Wenzhou People’s Hospital from January 2017 to December 2020 in this study. This retrospective study was approved by the Ethical Committee of FUSCC and Wenzhou People’s Hospital. All procedures were carried out in accordance with relevant guidelines.

### Inclusion and Exclusion Criteria

**Figure 1:**
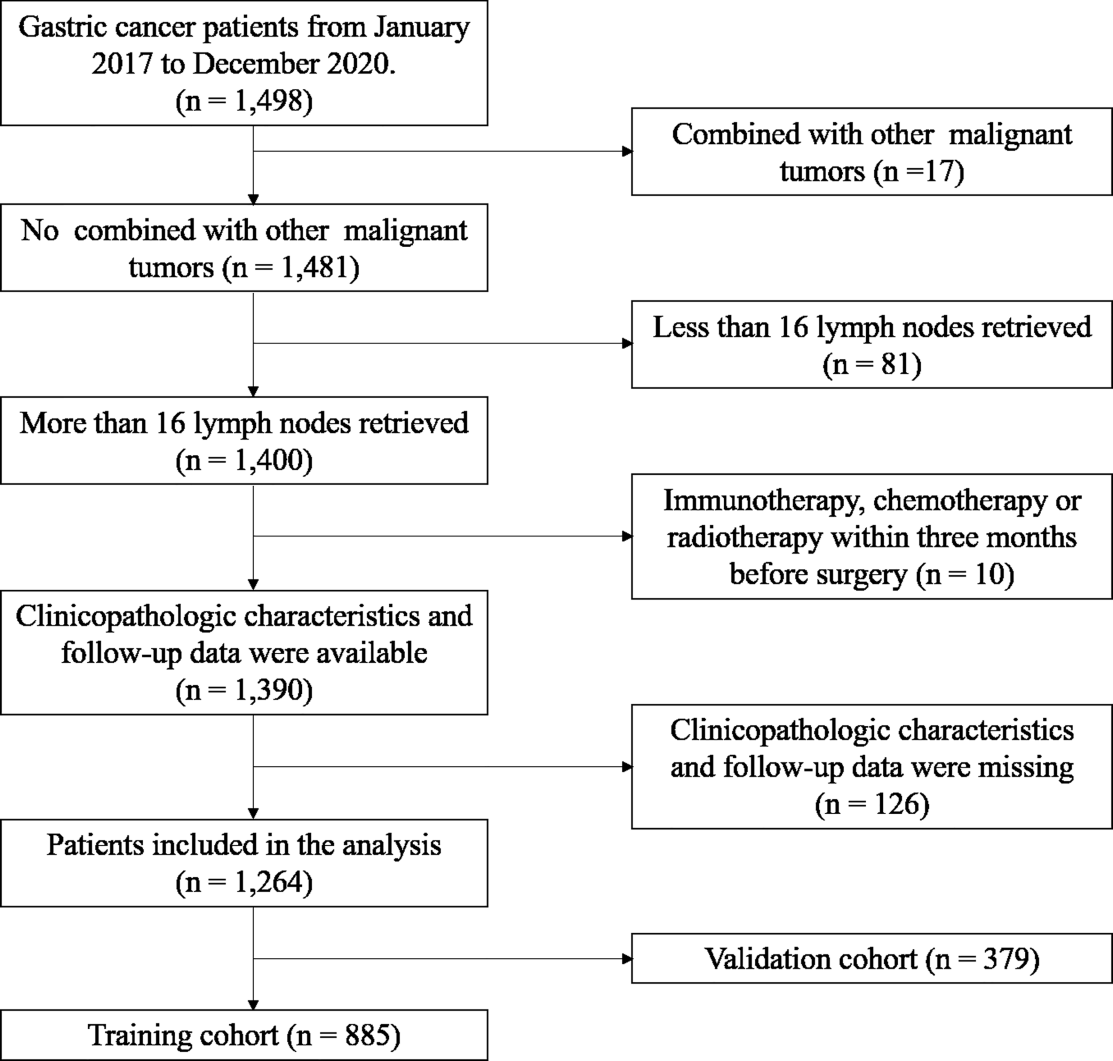
Flowchart of patient cohort definition.

The inclusion criteria were: (1) GC patients diagnosed by gastroscopy and CT examination; (2) No other forms of treatment, such as immunotherapy, chemotherapy or radiotherapy were received before treatment; (3) patients had no other malignant tumors; (4) With complete detection results of serum tumor markers, including CEA, CA19–9, CA125, CA50, CA242 and CA72-4. The exclusion criteria were: (1) radical resection was performed within 2 months after enrollment; (2) patients diagnosed with other malignancies or major diseases within 3 years prior to surgery (n=17); (3) patients received immunotherapy, chemotherapy or radiotherapy within 3 months before surgery (n=10); (4) Incomplete of clinicopathologic characteristics and follow-up data (n=126); (5) patients with distant organ metastasis, pregnancy or incomplete data were excluded. Finally, of these patients, 1264 eligible patients were identified for this study (**Figure 1**).

In the case-control study, in order to control confounding bias and exclude mutual metastasis of tumors, we did not include patients with other malignant tumors simultaneously, such as ovarian cancer, pancreatic cancer, colorectal cancer and other abdominal malignancies, for which peritoneal metastasis are prone to occur in the event of tumor recurrence and progression.

Current guidelines suggest that 16 or more lymph nodes (LNs) are required for the appropriate TMN staging of gastric cancer, the effect on survival of the minimum number of examined LNs in the different types of gastrectomy remains unclear. However, a retrospective analysis of 2,947 patients from a two-institution database in China showed that to maximize the survival benefit after radical total gastrectomy for gastric cancer, a minimum of 21 LNs should be removed (21). Therefore, in order to predict survival more accurately with TNM staging system, we excluded the cases with less than 16 detected lymph nodes.

### Study Patients

A total of 1264 GC patients were enrolled in our study, including 316 patients diagnosed with PD and 948 patients without PD, randomly matched at a ratio of 3:1. 885 patients were enrolled in the training cohort, including 221 patients with PD and 664 patients without PD, with a median age of 59 years. In addition, 379 patients were included in the validation cohort, including 95 patients with PD and 284 without PD, with a median age of 57 years. All patients were staged according to the Union for International Cancer Control (UICC) and American Joint Committee on Cancer (AJCC) TNM Staging Classification for CRC (Eighth Edition, 2016). The following baseline indicators were analyzed: age, sex, stage of TNM, preoperative tumor markers levels and some pathological conditions, including nerve infiltration and lympho-vascular invasion status. All patients underwent enhanced CT scan or magnetic resonance imaging (MRI) before surgery or treatment. Tumor size is measured by its longest diameter. The depth of invasion (clinical T stages) and lymphatic metastasis (clinical N stages) were obtained based on preoperative imaging examination. As shown in **Table 1**, we found no statistically significant differences in any clinicopathological features or tumor markers between two cohorts (p* value >0.05), indicating that the constitutions of the two groups were similar. Tumor size, cT stage, cN stage and serum tumor marker levels, including CEA, CA50, CA125, CA72-4, CA242 and CA19-9, were significantly different in both cohorts (P<0.05), indicating a significant correlation with PD.

**Table 1:** Correlation between peritoneal dissemination and clinicopathologic features [n (%)].

Group	Training cohort (n=885)				Validation cohort (n=379)				P*
	non.PD	PD	Standardize diff.	P-value	non.PD	PD	Standardize diff.	P-value	
Gender			0.03 (-0.12, 0.18)	0.721			0.01 (-0.22, 0.24)	0.933	0.965
Male	442 (66.57%)	150 (67.87%)			190 (66.90%)	64 (67.37%)			
Female	222 (33.43%)	71 (32.13%)			94 (33.10%)	31 (32.63%)			
Depth of invasion			2.07 (1.79, 2.35)	<0.001			4.91 (4.31, 5.52)	<0.001	<0.001
Mucosa(T1a)	119 (17.92%)	1 (1.56%)			57 (20.07%)	0 (0.00%)			
Submucosa(T1b)	158 (23.80%)	2 (3.12%)			85 (29.93%)	0 (0.00%)			
muscularis propria(T2)	226 (34.04%)	1 (1.56%)			117 (41.20%)	0 (0.00%)			
Within serosa (T3)	136 (20.48%)	36 (56.25%)			25 (8.80%)	7 (36.84%)			
Beyond serosa (T4a-4b)	25 (3.77%)	24 (37.50%)			0 (0.00%)	12 (63.16%)			
Lymphatic metastasis			0.93 (0.67, 1.19)	<0.001			1.40 (0.94, 1.87)	<0.001	0.200
N0	326 (49.10%)	9 (14.06%)			137 (48.24%)	1 (5.00%)			
N1(1-2)	90 (13.55%)	8 (12.50%)			47 (16.55%)	3 (15.00%)			
N2(3-6)	108 (16.27%)	11 (17.19%)			35 (12.32%)	1 (5.00%)			
N3-N4(≥7)	140 (21.08%)	36 (56.25%)			65 (22.89%)	15 (75.00%)			
vascular cancer embolus			0.81 (0.55, 1.07)	<0.001			0.57 (0.13, 1.02)	0.019	0.679
Absent	349 (53.12%)	11 (17.19%)			142 (50.35%)	5 (23.81%)			
Present	308 (46.88%)	53 (82.81%)			140 (49.65%)	16 (76.19%)			
Lympho-vascular invasion			0.84 (0.58, 1.10)	<0.001			1.14 (0.68, 1.61)	<0.001	0.837
Absent	332 (50.08%)	9 (14.06%)			139 (49.12%)	1 (5.00%)			
Present	331 (49.92%)	55 (85.94%)			144 (50.88%)	19 (95.00%)			
Perineural infiltration			0.54 (0.28, 0.80)	<0.001			0.64 (0.19, 1.10)	0.009	0.982
Absent	363 (55.34%)	19 (29.69%)			155 (54.96%)	5 (25.00%)			
Present	293 (44.66%)	45 (70.31%)			127 (45.04%)	15 (75.00%)			
Age			0.02 (-0.13, 0.17)	0.801			0.37 (0.13, 0.60)	0.003	0.081
≤60	318 (47.89%)	108 (48.87%)			154 (54.23%)	68 (71.58%)			
>60	346 (52.11%)	113 (51.13%)			130 (45.77%)	27 (28.42%)			
CEA			0.36 (0.21, 0.51)	<0.001			0.39 (0.16, 0.63)	<0.001	0.393
Negative(<5.2 ng/ml)	578 (87.05%)	161 (72.85%)			243 (85.56%)	66 (69.47%)			
Positive(≥5.2 ng/ml)	86 (12.95%)	60 (27.15%)			41 (14.44%)	29 (30.53%)			
CA50			0.51 (0.35, 0.66)	<0.001			0.35 (0.11, 0.58)	0.001	0.758
Negative(<25 U/ml)	630 (94.88%)	171 (78.08%)			264 (92.96%)	75 (81.52%)			
Positive(≥25 U/ml)	34 (5.12%)	48 (21.92%)			20 (7.04%)	17 (18.48%)			
CA125			0.73 (0.53, 0.93)	<0.001			0.73 (0.45, 1.01)	<0.001	0.123
Negative(<35 U/ml)	642 (96.69%)	84 (71.79%)			282 (99.30%)	48 (77.42%)			
Positive(≥35 U/ml)	22 (3.31%)	33 (28.21%)			2 (0.70%)	14 (22.58%)			
CA72-4			0.64 (0.48, 0.80)	<0.001			0.47 (0.23, 0.71)	<0.001	0.626
Negative(<6.9 U/ml)	556 (83.73%)	122 (55.71%)			236 (83.10%)	59 (62.77%)			
Positive(≥6.9 U/ml)	108 (16.27%)	97 (44.29%)			48 (16.90%)	35 (37.23%)			
CA242			0.46 (0.30, 0.61)	<0.001			0.36 (0.12, 0.59)	0.001	0.281
Negative(<20 U/ml)	615 (92.62%)	167 (76.61%)			255 (89.79%)	72 (76.60%)			
Positive(≥20 U/ml)	49 (7.38%)	51 (23.39%)			29 (10.21%)	22 (23.40%)			
Diameter			0.73 (0.47, 0.99)	<0.001			0.57 (0.11, 1.02)	0.048	0.344
<2cm	186 (28.01%)	2 (3.12%)			69 (24.30%)	1 (5.00%)			
≥2cm	478 (71.99%)	62 (96.88%)			215 (75.70%)	19 (95.00%)			
CA19-9			0.43 (0.27, 0.58)	<0.001			0.45 (0.21, 0.68)	<0.001	0.814
Negative(<27 U/ml)	561 (84.49%)	147 (66.52%)			239 (84.15%)	62 (65.26%)			
Positive(≥27 U/ml)	103 (15.51%)	74 (33.48%)			45 (15.85%)	33 (34.74%)			

PD, peritoneal dissemination. cT satge (clinical T stage), depth of invasion. cN stage (clinical N stage), lymphatic metastasis. Standardize diff., standard difference. P*, the difference between the training cohort and the validation cohort.

### Statistical Analysis

Comparisons between groups were performed by Chi-square test or Fisher’s exact test. Risk factors related to PD in gastric cancer were determined by Logistic regression analysis. The area under ROC curve was used to evaluate the diagnostic value of tumor markers, and the optimal cut-off value was obtained by the ROC curve and Youden index. A nomogram was made as a prediction model of PD incidence, and the accuracy of the prediction was verified by ROC analysis and calibration plot. Statistical analysis was performed using SPSS 25.0 and R software (version x64). Differences with P<0.05 were considered statistically significant.

## Results

### Univariate and Multivariate Analysis for PD

Risk factors associated with PD in gastric cancer were determine by using logistic regression analysis (**Table 2**). According to univariate analysis, the following relevant factors found to be associated with PD: tumor diameter (OR=12.06, p<0.0006), depth of invasion (cT stage) (OR=14.55, p=0.0006), lymph node metastases (cN stage) (OR=5.89, p<0.0001), and serum tumor markers, including CEA (OR=2.50, p<0.0001), CA125 (OR=11.46, p<0.0001), CA72-4 (OR=4.09, p<0.0001), CA19-9 (OR=2.74, p<0.0001), CA50 (OR=5.20, p<0.0001) and CA242 (OR=3.83, p<0.0001). Additionally, multivariate analysis illustrated that depth of invasion (cT stages) (OR=9.233, p=0.043) and serum tumor markers of CA125 (OR=4.582, p<0.0001) and CA72-4 (OR=4.674, p<0.0001) were independent risk factors for PD.

**Table 2:** Logistic regression analysis of the risk factors for peritoneal dissemination.

	univariate analysis		multivariate analysis	
	OR (95%CI)	P value	OR (95%CI)	P value
Gender	0.94 (0.68, 1.30)	0.7206	/	/
Age (years)	0.96 (0.71, 1.30)	0.8012	/	/
Diameter (cm)	12.06 (2.92, 49.82)	0.0006	0.810 (0.136, 4.828)	0.363
Depth of invasion	14.55 (4.52, 46.86)	<0.0001	9.233 (1.073, 79.49)	0.043
Lymphatic metastasis	5.89 (2.87, 12.12)	<0.0001	1.983 (0.624, 6.305)	0.246
CEA (ng/mL)	2.50 (1.72, 3.64)	<0.0001	1.037 (0.295, 3.646)	0.681
CA125 (U/ml)	11.46 (6.38, 20.59)	<0.0001	4.582 (2.526, 8.312)	<0.0001
CA72-4 (U/mL)	4.09 (2.92, 5.73)	<0.0001	4.674 (2.673, 8.173)	<0.0001
CA19-9 (U/mL)	2.74 (1.93, 3.89)	<0.0001	0.356 (0.041, 3.050)	0.346
CA50 (U/mL)	5.20 (3.25, 8.33)	<0.0001	1.746 (0.265, 11.502)	0.562
CA242 (U/mL)	3.83 (2.50, 5.88)	<0.0001	1.573 (0.536, 4.613)	0.409

OR, odds ratio; 95% CI, confidence interval.

### ROC Curve of Significant Risk Factors

**Figure 2:**
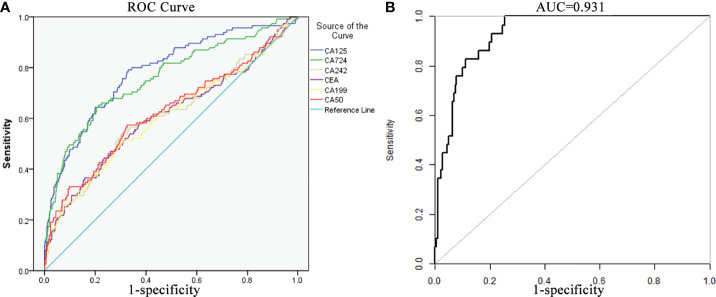
Receiver operating curve (ROC) curves of independent risk factors. **(A)** ROC curve of the important risk factors, including 6 serum tumor markers (CA125, CA724, CA242, CEA, CA199 and CA50). **(B)** ROC curve of the multivariate logistic regression model (AUC of the curve was 0.931, 95% CI: 0.900–0.960).

ROC curves were drawn to show the correlation between the risk of PD and tumor markers (CEA, CA125, CA72-4, CA19-9, CA242 and CA50) in GC patients (**Figure 2A**). The AUC of CA125 was 0.785 (95% CI: 0.738–0.833) with specificity and sensitivity values of 67.02% and 79.5%, respectively. The AUC of CA72-4 was 0.704 (95% CI: 0.663–0.745) with specificity and sensitivity values of 78.6% and 52.5%, respectively. The AUC of the combined factors was 0.931 (95% CI: 0.900–0.960) with specificity and sensitivity values of 82.2% and 90.5%, respectively, demonstrating good consistency and reliability in the predictive value of PD (**Figure 2B**). According to the ROC analysis for continuous predictors, the optimum cutoff values of CA125 and CA72-4 were 12.29 U/ml and 4.81 U/ml, respectively. By calculating and comparing their diagnostic value, we found that use of the optimal boundary value can significantly improve the sensitivity of PD diagnosis.

### The Construction of Nomogram for Predicting PD of GC

**Figure 3:**
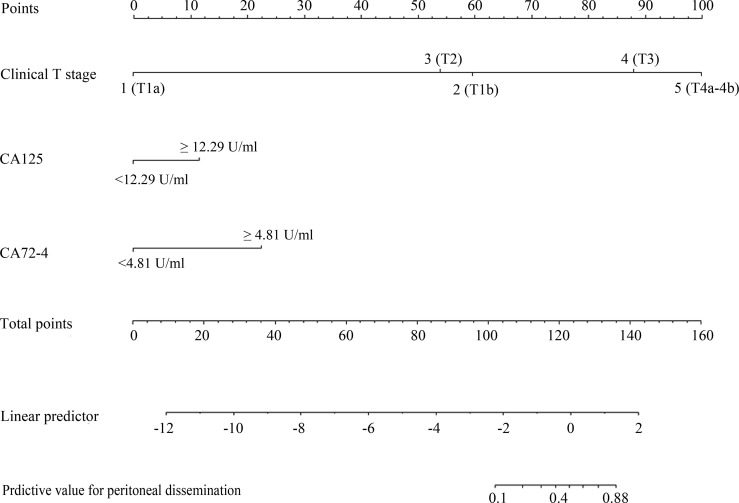
A nomogram composed of the independent risk factors to predict the probability of peritoneal dissemination for patients with gastric cancer. The risk value of PD was calculated by drawing a vertical line to the point to the axis on each of the variables. Add the points of each variable and locate them on the total point line, and then the individual predictive value for PD was obtained by projecting the vertical line from the total point line to the bottom scale of the prediction probability.

Based on these risk factors of PD incidence, a nomogram was further constructed (**Figure 3**). For each patient, points were based on the score of these clinicopathological risk factors on the underlying scale (clinical T stage and tumor markers of CA125 and CA72-4). By adding the points of each variable on the score scale, the total points projected to the bottom scale represent the probability of PD. The total score was 137 points as calculated from the nomogram with a probability of PD in GC of 69–80%. The concordance index (C-index) in the nomogram was 0.931 (95% CI: 0.900–0.960) with a sensitivity and specificity of 82.6% and 74.5%, respectively, indicating high accuracy and sensitivity of the prediction model (**Figure 2B**).

In addition, compared with previous studies, we also found that the C-index of our prediction model was significantly higher than that of previous studies. Zheng Z et al. constructed a nomogram of patients with early gastric cancer (EGC) based on age, depth of invasion, tumor differentiation and ulcer presence, with an AUC of 0.860 (95%CI: 0.809-0.912) (22). Ahmad et al. predicted the incidence of lymph node metastasis in EGC patients based on lympho-vascular infiltration and depth of invasion, and the AUC value was 0.684 (95%CI: 0.648-0.746) (23). Similarly, Holscher and Li Hua et al. also constructed a prediction model for lymph node metastasis in early gastric cancer patients, with AUC values of 0.738 (95%CI: 0.673-0.785) and 0.795 (95%CI: 0.723-0.858), respectively (24, 25). The results show that the current model has high prediction accuracy. Therefore, we have good reason to believe that this predictive model will contribute to the prognosis assessment and optimal treatment strategies of patients with gastric cancer.

**Figure 4:**
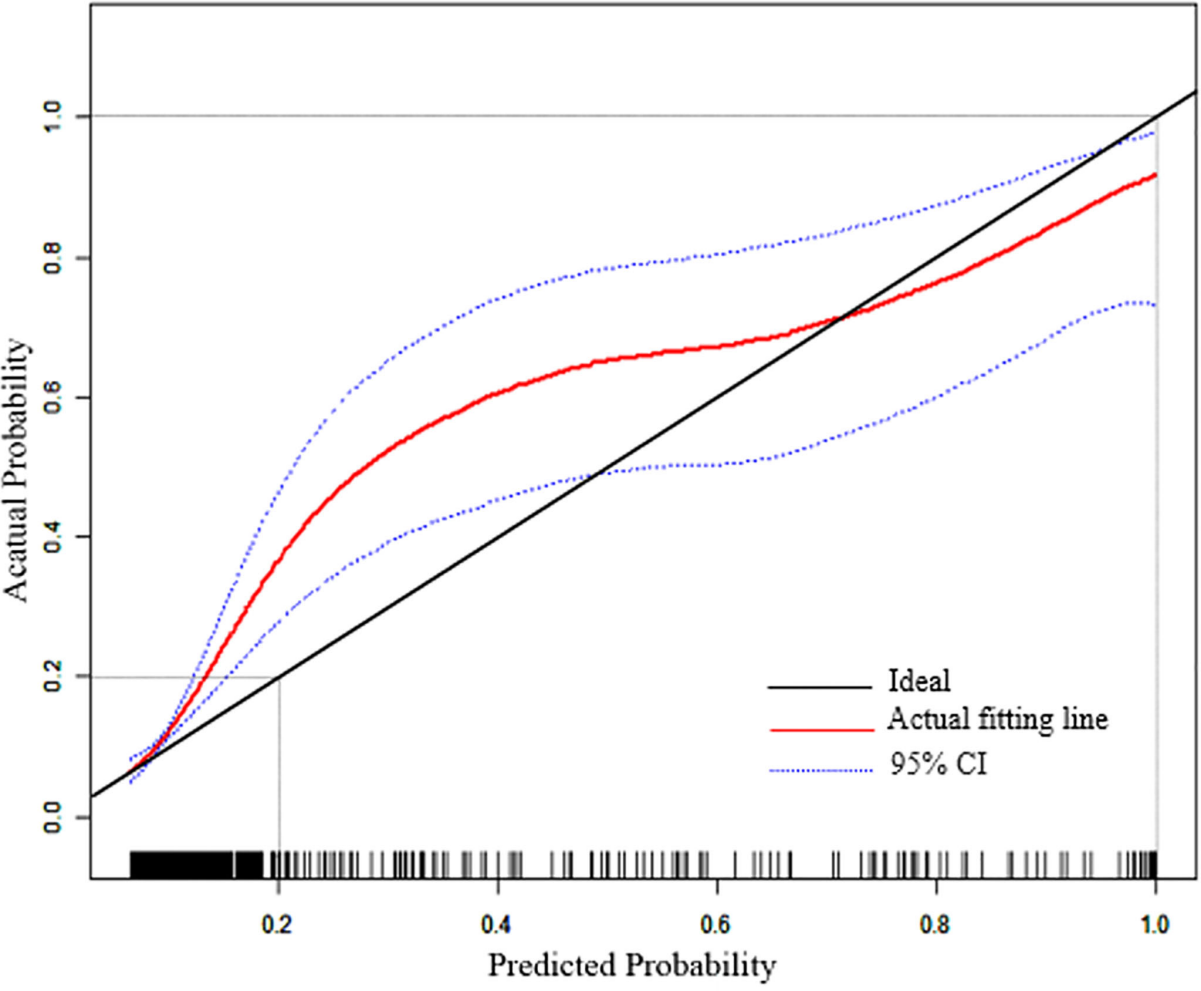
Calibration plot of the nomogram model. The solid line (black) is the reference line and indicates that the predicted value is equal to the actual value. The dashed line (red) represents the actual nomogram curve fitting line, whereas the dashed line (blue) represents the 95% CI.

In addition, a calibration plot was drawn to verify the accuracy of the prediction model (**Figure 4**). The x-coordinate is the predicted incidence of PD events, and the y-coordinate is the actual prediction of PD. The solid line (black) is the reference line and indicates that the predicted value is equal to the actual value. The dashed line (red) represents the actual nomogram curve fitting line, whereas the dashed line (blue) represents the 95% CI. The black solid line was close to the red dotted line, indicating the good predictive ability of this model.

## Discussion

As one of most commonly observed malignant tumors, gastric cancer patients with peritoneal dissemination tend to have a poor prognosis, with a median survival of 4 months, and 5-year survival rate is only 2% (10, 11). GC patients with peritoneal dissemination cannot receive radical surgery. Therefore, the accurate prediction of PD is of great significance in developing optimal treatment strategies for GC patients.

At present, imaging examination plays an indispensable role in screening and diagnosis in tumor disease. Although specificity and accuracy are high, the sensitivity is relatively low. In a retrospective analysis of 498 patients, Kim et al. (26) showed that the specificity and sensitivity of preoperative computed tomography (CT) for the diagnosis of PD were 98.9%(440 of 445) and 28.3%(15 of 53), respectively. In addition, Kayaalp et al. (27) evaluated preoperative peritoneal metastases in 118 patients with GC compared with the surgical results, and found that the sensitivity of CT in PD diagnosis was only 13%. The detection sensitivity of CT to PD detection was affected by the size of nodules. Nodules smaller than 0.5 cm show only 11% sensitivity on CT, leading to a high rate of missed diagnosis (17). Laparoscopic exploration is highly accurate in the diagnosis of PD, but it is an invasive procedure.

Serum tumor markers are important pathological factors for the clinical diagnosis and prognostic evaluation of tumors, and are widely accepted as convenient and quick methods. In recent years, many studies have confirmed that serum tumor markers, particularly CEA, CA125, CA19-9 and CA72-4, have important value in the diagnosis and progress monitoring of gastrointestinal malignancies. However, the sensitivity of individual tumor markers was not satisfactory (19, 28, 29). Previous studies have used a variety of biomarker combinations to improve the sensitivity of GC diagnosis. Yang AP et al. (30) reported that the sensitivities for GC diagnosis of CA125, CA19–9, CA72–4 and CEA were 38.7%, 31.1%, 33.0% and 25.5%, respectively. However, the sensitivity increased to 66.0% when the four serum markers were used in combination. Shigenobu Emoto et al. (19) reported a similar result. Specifically, the sensitivities of CA125, CA19–9, CA72–4 and CEA were 36.3%, 46.1%, 44.9% and 18.6%, respectively. The sensitivity was 78.4% for the combination of all 4 markers. In view of this finding, we aimed to identify a simple, highly sensitive and noninvasive prediction method.

In this study, we investigated the value of clinicopathological features and serum tumor markers as diagnostic markers of PD in gastric cancer. The risk factors associated with PD were assessed by logistic regression analysis. Multivariate analysis illustrated that depth of invasion and CA125 and CA72-4 serum markers were independent risk factors for PD. CA125, also known as MUC16, is a glycoprotein antigen of transmembrane mucin that is associated with many malignant tumors with poorer prognosis. Previous studies have shown that CA125 is a powerful predictor of PD in GC patients and has a high sensitivity (19, 31–33). CA72–4 is a high molecular weight glycoprotein antigen with elevated expression in many cancers and has high specificity for GC diagnosis (34–37). In a retrospective clinical study of 102 patients with peritoneal dissemination undergoing chemotherapy, CA72–4 was found to be second only to CA125 in sensitivity as a marker for peritoneal dissemination (19). In addition, Tong et al. (38) verified that CA72-4 is an independent risk factor for prognosis and can be used to predict TNM staging in locally advanced GC patients undergoing radical resection.

In recent years, nomogram has been widely used in diagnosis and prediction of various cancers (39–41). Compared to the traditional TNM staging system, nomograms provide more accurate information for the description of lesions. In this study, a nomogram based on clinicopathological factors (tumor size, TNM stage, vascular cancer embolus status, lymph node and nerve invasion status) and tumor marker level has predictive effect on PD. First, we use the ROC curve to determine the optimum cutoff values. Then, a nomogram was constructed according to the related risk factors for PD. In addition, ROC curve and calibration plot were drawn to verify the accuracy of this prediction model. The C-index of our prediction model is 0.931, which is significantly higher than some previous prediction models (22–25), indicated a better prediction accuracy for PD in GC. Therefore, the nomogram constructed in our study can effectively predict incidence of PD in gastric cancer patients. Through which surgeons could make more accurate preoperative diagnoses and optimal treatment strategies for GC patients.

However, there are several limitations to our study. First, it was a retrospective study conducted at a single center, and the sample size was not large enough. Thus, some bias potentially occurred during the analysis. Second, blood levels of the tumor-related inflammatory markers, such as serum neutrophils, lymphocytes, platelets and C-reactive protein were not included in this study. Furthermore, there was no correlation analysis of the prognostic profiles in our study.

In conclusion, the nomogram presented in this study provides an efficient and reliable prediction model for the incidence of PD in gastric cancer patients undergoing radical resection. It is hoped hope that our predictive model can provide a useful tool for the prognosis assessment and personalized treatment selection of GC patients.

## Conclusion

In this study, clinical pathological features and preoperative serum tumor markers were used to predict the incidence of PD in gastric cancer patients, and to establish a risk assessment model for GC. The results showed that CA125 > 12.29 U/ml and CA72-4 > 4.81 U/ml were risk factors for PD. The prediction model constructed based on preoperative tumor invasion and CA125 and CA72–4 serum markers exhibit high specificity and accuracy for the incidence of PD. We expect that the results of our study can provide clinical value for the preoperative evaluation of GC patients and the selection of individualized treatment for GC patients.

## Data Availability Statement

The original contributions presented in the study are included in the article/**Supplementary Material**. Further inquiries can be directed to the corresponding author.

## Ethics Statement

The studies involving human participants were reviewed and approved by the Ethical Committee of University Shanghai Cancer Center(FUSCC). The patients/participants provided their written informed consent to participate in this study. Written informed consent was obtained from the individual(s) for the publication of any potentially identifiable images or data included in this article.

## Author Contributions

DB and YH designed the study and wrote the manuscript with contributions from all authors. SC and KL collected clinical data. DB, ZY and SC analyzed the data. YH reviewed the manuscript. All authors read and approved the final version of the paper.

## Funding

The authors are thankful to the National Nature Science Foundation of China (Grant Nos. 81972213), Fudan University Shanghai Cancer Center for Outstanding Youth Scholars Foundation (YJYQ201803). The sponsors have no role in study design, in the collection, analysis and interpretation of data, in the writing of the report, and in the decision to submit the article for publication.

## Conflict of Interest

The authors declare that the research was conducted in the absence of any commercial or financial relationships that could be construed as a potential conflict of interest.

## Publisher’s Note

All claims expressed in this article are solely those of the authors and do not necessarily represent those of their affiliated organizations, or those of the publisher, the editors and the reviewers. Any product that may be evaluated in this article, or claim that may be made by its manufacturer, is not guaranteed or endorsed by the publisher.

## References

[1] Bray F Ferlay J Soerjomataram I Siegel RL Torre LA Jemal A. Global Cancer Statistics 2018: GLOBOCAN Estimates of Incidence and Mortality Worldwide for 36 Cancers in 185 Countries. CA Cancer J Clin (2018) 68:394–424. doi: 10.3322/caac.21492 30207593

[2] Shen L Shan SY Hu HM Price TJ Sirohi B Yeh KH. Management of Gastric Cancer in Asia: Resource-Stratified Guidelines. Lancet Oncol (2013) 14:e535–47. doi: 10.1016/S1470-2045(13)70436-4 24176572

[3] Kanda M Kobayashi D Tanaka C Iwata N Yamada S Fujii T. Adverse Prognostic Impact of Perioperative Allogeneic Transfusion on Patients With Stage II/III Gastric Cancer. Gastric Cancer (2016) 19:255–63. doi: 10.1007/s10120-014-0456-x 25563579

[4] Hartgrink HH Jansen EPM van Grieken NC van de Velde CJ. Gastric Cancer. Lancet (2009) 374:477–90. doi: 10.1016/S0140-6736(09)60617-6 PMC4613761 19625077

[5] Siegel R Naishadham D Jemal A. Cancer Statistics, 2012. CA Cancer J Clin (2012) 62:10–29. doi: 10.3322/caac.20138 22237781

[6] Shiozaki HE Elimova E Slack RS Chen HC Staerkel GA Sneige N. Prognosis of Gastric Adenocarcinoma Patients With Various Burdens of Peritoneal Metastases. J Surg Oncol (2016) 113:29–35. doi: 10.1002/jso.24087 26603684

[7] Badgwell B Roy-Chowdhuri S Chiang YJ Matamoros A Blum M Fournier K. Long-Term Survival in Patients With Metastatic Gastric and Gastroesophageal Cancer Treated With Surgery. J Surg Oncol (2015) 111(7):875–81. doi: 10.1002/jso.23907 25872485

[8] Kaya DM Nogueras-González GM Harada K Amlashi FG Roy-Chowdhuri S Estrella JS. Risk of Peritoneal Metastases in Patients Who had Negative Peritoneal Staging and Received Therapy for Localized Gastric Adenocarcinoma. J Surg Oncol (2018) 117(4):678–84. doi: 10.1002/jso.24912 PMC5878692 29205363

[9] Sadeghi B Arvieux C Glehen O Beaujard AC Rivoire M Baulieux J. Peritoneal Carcinomatosis From non-Gynecologic Malignancies: Results of the EVOCAPE 1 Multicentric Prospective Study. Cancer (2000) 88:358–63. doi: 10.1002/(sici)1097-0142(20000115)88:2<358:aid-cncr16>3.0.co;2-o 10640968

[10] Thomassen I van Gestel YR van Ramshorst B Luyer MD Bosscha K Nienhuijs. Peritoneal Carcinomatosis of Gastric Origin: A Population-Based Study on Incidence, Survival and Risk Factors. Int J Cancer (2014) 134:622–8. doi: 10.1002/ijc.28373 23832847

[11] Kanda M Kodera Y. Molecular Mechanisms of Peritoneal Dissemination in Gastric Cancer. World J Gastroenterol (2016) 22(30):6829–40. doi: 10.3748/wjg.v22.i30.6829 PMC4974582 27570420

[12] Ajani JA D'Amico TA Almhanna K Bentrem DJ Chao J Das P. Gastric Cancer, Version 3.2016, NCCN Clinical Practice Guidelines in Oncology. J Natl Compr Cancer Netw (2016) 14:1286–312. doi: 10.6004/jnccn.2016.0137 27697982

[13] JGCA. Japanese Gastric Cancer Treatment Guidelines 2014 (Ver. 4). In: Gastric Cancer, 465 Kajii cho, Kawaramachihirokoji, Kamigyo ku, Kyoto, 602-8566 Japan: Japanese Gastric Cancer Association. vol. 20. (2017). p. 1–19. doi: 10.1007/s10120-016-0622-4 PMC5215069 27342689

[14] Chia DKA So JBY. Recent Advances in Intra-Peritoneal Chemotherapy for Gastric Cancer. J Gastric Cancer (2020) 20(2):115–26. doi: 10.5230/jgc.2020.20.e15 PMC7311211 32595996

[15] Burbidge S Mahady K Naik K. The Role of CT and Staging Laparoscopy in the Staging of Gastric Cancer. Clin Radiol (2013) 68(3):251–5. doi: 10.1016/j.crad.2012.07.015 22985749

[16] Yang Q-M Bando E Kawamura T Tsukiyama G Nemoto M Yonemura Y. The Diagnostic Value of PET-CT for Peritoneal Dissemination of Abdominal Malignancies. Gan To Kagaku Ryoho (2006) 33:1817–21. 17212117

[17] Koh JL Yan TD Glenn D DL M. Evaluation of Preoperative Computed Tomography in Estimating Peritoneal Cancer Index in Colorectal Peritoneal Carcinomatosis. Ann Surg Oncol (2009) 16:327–33. doi: 10.1245/s10434-008-0234-2 19050972

[18] Yan TD Sim J Morris DL. Selection of Patients With Colorectal Peritoneal Carcinomatosis for Cytoreductive Surgery and Perioperative Intraperitoneal Chemotherapy. Ann Surg Oncol (2006) 14:1807–17. doi: 10.1245/s10434-007-9350-7 17342564

[19] Emoto S Ishigami H Yamashita H Yamaguchi H Kaisaki S Kitayama J. Clinical Significance of CA125 and CA72-4 in Gastric Cancer With Peritoneal Dissemination. Gastric Cancer (2012) 15:154–61. doi: 10.1007/s10120-011-0091-8 21892754

[20] Hackbarth JS Murata K Reilly WM Algeciras-Schimnich A. Performance of CEA and CA19-9 in Identifying Pleural Effusions Caused by Specific Malignancies. Clin Biochem (2010) 43:1051–5. doi: 10.1016/j.clinbiochem.2010.05.016 20529669

[21] Huang C Lu J Zheng C Li P. How Many Lymph Nodes Should be Removed to Define an Optimal D2 Lymphadenectomy for Gastric Cancer in the Modern Era? An Analysis of 2,947 Patients From a Two-Institution Database in China. J Clin Oncol (2016) 34:4051. doi: 10.1200/JCO.2016.34.15_suppl.4051 27528720

[22] Zheng Z Zhang Y Zhang L Li Z Wu X Liu Y. A Nomogram for Predicting the Likelihood of Lymph Node Metastasis in Early Gastric Patients. BMC Cancer (2016) 16:92. doi: 10.1186/s12885-016-2132-5 26873736 PMC4751748

[23] Ahmad R Namrata S Schmidt BH Hong TS Wo JY Kwak EL. Predictors of Lymph Node Metastasis in Western Early Gastric Cancer. J Gastrointest Surg (2016) 20(3):531–8. doi: 10.1007/s11605-015-2945-6 26385006

[24] Holscher AH Drebber U Monig SP Schulte C Vallbohmer D Bollschweiler E. Early Gastric Cancer: Lymph Node Metastasis Starts With Deep Mucosal Infiltration. Ann Surg Oncol (2009) 250:791–7. doi: 10.1097/SLA.0b013e3181bdd3e4 19809298

[25] Li H Lu P Lu Y Liu C Xu H Wang S. Predictive Factors of Lymph Node Metastasis in Undifferentiated Early Gastric Cancers and Application of Endoscopic Mucosal Resection. Surg Oncol (2010) 19:221–6. doi: 10.1016/j.suronc.2009.05.006 20471826

[26] Kim SJ Kim HH Kim YH Hwang SH Lee HS Park DJ. Peritoneal Metastasis: Detection With 16- or 64-Detector Row CT in Patients Undergoing Surgery for Gastric Cancer. Radiology (2009) 253(2):407–15. doi: 10.1148/radiol.2532082272. Epub 2009 Sep 29 19789243

[27] Kayaalp C Arda K Orug T Ozcay N. Value of Computed Tomography in Addition to Ultrasound for Preoperative Staging of Gastric Cancer. Eur J Surg Oncol (2002) 28(5):540–3. doi: 10.1053/ejso.2002.1296 12217308

[28] Gadde R Tamariz L Hanna M Avisar E Livingstone A Franceschi D. Metastatic Gastric Cancer (MGC) Patients: Can We Improve Survival by Metastasectomy? A Systematic Review and Meta-Analysis. J Surg Oncol (2015) 112(1):38–45. doi: 10.1002/jso.23945 26074130

[29] Mohri Y Tanaka K Ohi M Saigusa S Yasuda H Toiyama Y. Identification of Prognostic Factors and Surgical Indications for Metastatic Gastric Cancer. BMC Cancer (2014) 6. 14:409. doi: 10.1186/1471-2407-14-409 PMC4057566 24906485

[30] Yang AP Liu J Lei HY Zhang QW Zhao L Yang G-H. A72-4 Combined With CEA, CA125 and CAl9-9 Improves the Sensitivity for the Early Diagnosis of Gastric Cancer. Clin Chim Acta (2014) 437:183–6. doi: 10.1016/j.cca.2014.07.034 25086284

[31] Nakata B Chung KHYS Kato Y Yamashita Y Maeda K Onoda N. Serum CA125 Level as a Predictor of Peritoneal Dissemination in Patients With Gastric Carcinoma. Cancer Cancer (1998) 83(12):2488–92. doi: 10.1002/(sici)1097-0142(19981215)83:12<2488::aid-cncr12>3.0.co;2-1 9874453

[32] Hwang GI Yoo C Sohn BH Shin JH Park YL Kim HD. Predictive Value of Preoperative Serum CEA, CA19-9 and CA125 Levels for Peritoneal Metastasis in Patients With Gastric Carcinoma. Cancer Res Treat (2004) 36:178–81. doi: 10.4143/crt.2004.36.3.178 PMC2855081 20396541

[33] Fujimura T Kinami S Ninomiya I Kitagawa H Fushida S Nishimura G. Diagnostic Laparoscopy, Serum CA125, and Peritoneal Metastasis in Gastric Cancer. Endoscopy (2002) 34:569–74. doi: 10.1055/s-2002-33228 12170412

[34] Kim DH Oh SJ Oh CA Choi MG Noh JH Sohn TS. The Relationships Between Perioperative CEA, CA 19-9, and CA 72-4 and Recurrence in Gastric Cancer Patients After Curative Radical Gastrectomy. J Surg Oncol (2011) 104(6):585–91. doi: 10.1002/jso.21919 21695697

[35] Mattar R Alves de Andrade CR DiFavero GM Gama-Rodrigues JJ Laudanna AA. Preoperative Serum Levels of CA 72-4, CEA, CA 19-9, and Alpha-Fetoprotein in Patients With Gastric Cancer. Rev Hosp Clin Fac Med Sao Paulo (2002) 57(3):89–92. doi: 10.1590/s0041-87812002000300001 12118264

[36] Goral V Yesilbagdan H Kaplan A Sit D. Evaluation of CA 72-4 as a New Tumor Marker in Patients With Gastric Cancer. Hepatogastroenterology (2007) 54(76):1272–5. 17629087

[37] Sougioultzis S Syrios J Xynos ID Bovaretos N Kosmas C Sarantonis J. Palliative Gastrectomy and Other Factors Affecting Overall Survival in Stage IV Gastric Adenocarcinoma Patients Receiving Chemotherapy: A Retrospective Analysis. Eur J Surg Oncol (2011) 37(4):312–8. doi: 10.1016/j.ejso.2011.01.019 21300519

[38] Tong Y Zhao Y Shan Z Zhang J. CA724 Predicts Overall Survival in Locally Advanced Gastric Cancer Patients With Neoadjuvant Chemotherapy. BMC Cancer (2021) 21:4. doi: 10.1186/s12885-020-07666-8 33402124 PMC7786973

[39] Li Y Jia H Yu W Xu Ye Li X Li Q. Nomograms for Predicting Prognostic Value of Inflammatory Biomarkers in Colorectal Cancer Patients After Radical Resection. Int J Cancer (2016) 139(1):220–31. doi: 10.1002/ijc.30071 26933932

[40] Wang Y Li J Xia Y Gong R Wang K Yan Z. Prognostic Nomogram for Intrahepatic Cholangiocarcinoma After Partial Hepatectomy. J Clin Oncol (2013) 31:1188–95. doi: 10.1200/JCO.2012.41.5984 23358969

[41] Hu S Gan W Qiao L Ye C Wu D Liao B. A New Prognostic Algorithm Predicting HCC Recurrence in Patients With Barcelona Clinic Liver Cancer Stage B Who Received PA-TACE. Front Oncol (2021) 21. 11:742630. doi: 10.3389/fonc.2021.742630 PMC8566809 34745962

